# 612 Racial Differences in Post-burn Hypertrophic Scarring Incidence with Glucocorticoid Effectiveness

**DOI:** 10.1093/jbcr/iraf019.241

**Published:** 2025-04-01

**Authors:** Joshua Lewis, Gengi Kleto, Bethel Desta, Blancheneige Beohon, Raven Hollis, Philong Nguyen, George Golovko, Juquan Song

**Affiliations:** University of Texas Medical Branch; University of Texas Medical Branch; University of Texas Medical Branch; University of Texas Medical Branch; University of Texas Medical Branch; University of Texas Medical Branch; University of Texas Medical Branch; University of Texas Medical Branch

## Abstract

**Introduction:**

Hypertrophic scarring is a frequent complication after burn injuries, with notable variation in incidence and treatment response among racial groups. Glucocorticoids are commonly used to manage hypertrophic scars, but their effectiveness may differ by race. This study aims to 1) examine racial differences in hypertrophic scarring incidence among burn patients and 2) evaluate the clinical effectiveness of glucocorticoid treatments in reducing hypertrophic scarring in African Americans, Asians, and Whites one-year post-burn injury.

**Methods:**

Using the TriNetX Collaborative Network, we identified 633,708 burn patients aged ≥18 treated between 2013 and 2023. Patients were grouped by race: White, Black, Asian, American Indian, and Native Hawaiian. The incidence of hypertrophic scarring and the effectiveness of glucocorticoids were assessed at one month, six months, and one-year post-burn by calculating the relative risk ratio for each racial group, with White patients as the reference. Propensity score matching controlled for age, sex, and burn severity. Statistical significance was set at p< 0.05.

**Results:**

Among the burn patients in TriNetX database, 46,893 developed hypertrophic scarring, with 63.06% were White, 13.50% were Black or African American, 2.47% were Asian, 0.55% were Native Hawaiian, and 0.54% were American Indian. Among all patients with burns, African Americans (p< 0.0001), Asian (p< 0.0001), and Native Hawaiian ((p< 0.05) had a significantly increased risk for hypertrophic scarring compared to White burn patients. However, Native Hawaiian had significantly decreased risk for hypertrophic scarring compared to White burn patients (p< 0.05). When assessing effectiveness in glucocorticoids, hydrocortisone and triamcinolone statistically increased risk for hypertrophic scarring in Black and Asian patients compared to White burn patients (p< 0.05). However, dexamethasone decreased risk for hypertrophic scarring in African American patients but not Asians (p=0.0518) and methylprednisolone decreased risk for hypertrophic scarring in Asian patients but not African Americans (p=0.1537).

**Conclusions:**

This study reveals significant racial disparities in hypertrophic scarring following burn injuries and highlights the differential effectiveness of glucocorticoid treatments across racial groups. These findings emphasize the need for personalized treatment strategies to address scarring in burn patients from diverse racial backgrounds.

**Applicability of Research to Practice:**

The findings of this study have significant implications for the clinical management of hypertrophic scarring in burn patients. By identifying racial disparities in both the incidence of hypertrophic scarring and the differential effectiveness of glucocorticoid treatments, this research underscores the need for personalized, race-conscious treatment strategies.

**Funding for the Study:**

This research was supported by a Clinical and Translational Science Award (UL1 TR001439) from the National Center for Advancing Translational Sciences at the National Institutes of Health (NIH). The content is solely the responsibility of the authors and does not necessarily represent the official views of the NIH.

